# The value of regional and global CACS combined with SPECT MPI in detecting obstructive CAD: a retrospective real-world comparative study

**DOI:** 10.1186/s12872-023-03051-y

**Published:** 2023-01-11

**Authors:** Qi Jiang, Bao Liu, Le Yang, Yufeng Wang, Wenji Yu, Feifei Zhang, Yunmei Shi, Yuetao Wang

**Affiliations:** 1 Department of Nuclear Medicine, The Third Affiliated Hospital of Soochow University, No.185, Juqian Street, Changzhou, 213003 Jiangsu Province China; 2 Yangzhou University, Yangzhou, Jiangsu Province China

**Keywords:** Myocardial perfusion imaging, SPECT, Coronary artery calcium score, Coronary artery disease

## Abstract

**Objective:**

Previous studies have shown that global coronary artery calcium score (CACS) can improve single photon emission computerized tomography (SPECT) myocardial perfusion imaging (MPI) to detect obstructive coronary artery disease (CAD). Whether regional CACS can improve SPECT MPI to detect obstructive CAD remains unclear. The aim of this study was to verify whether regional CACS has additional diagnostic value for obstructive CAD in suspected patients, compared to SPECT MPI and global CACS.

**Methods:**

The study included 321 suspected CAD patients who underwent one-stop rest-stress SPECT MPI and low-dose computed tomography (CT) scan. All patients underwent coronary angiography within one month after examination. MPI images were visually analyzed by 2 experienced nuclear cardiologists. The regional CACS of left anterior descending coronary artery (LAD), left circumflex coronary artery (LCX), right coronary artery (RCA) and global CACS were calculated. Obstructive CAD was defined as ≥ 70% narrowing of the inner diameter of the LAD, LCX, RCA or their main branches and ≥ 50% narrowing of the left main coronary artery (LM).

**Results:**

Among the 321 patients, 86 (26.8%, 86/321) had obstructive CAD. With the increased in global and regional CACS, there was an increasing trend of patients with obstructive CAD (*P* for trend < 0.001). Regional CACS had a better diagnostic performance in RCA territories (AUC 0.856, *P* < 0.001) compared with LAD, LCX territories (AUC 0.690, 0.674, respectively). The AUC of combined regional CACS and MPI was significantly higher than that of MPI alone (0.735 vs. 0.600, *P* < 0.001). However, based on MPI, the AUC of combined regional CACS was not significantly higher than that of global CACS (0.735 vs. 0.732, *P* = 0.898). The sensitivity and specificity of regional CACS combined with MPI for detecting obstructive CAD were 64.0% and 72.8%, respectively.

**Conclusions:**

Regional CACS was effective in detecting obstructive CAD in RCA territory. Based on SPECT MPI, regional CACS improved the detection of obstructive CAD, but was not superior to global CACS.

## Introduction

A large number of people worldwide suffer from coronary artery disease (CAD). The main cause of CAD is stenosis caused by coronary atherosclerotic plaques. It is noteworthy that in patients with narrow coronary arteries > 70%, more than 80% have functional myocardial ischemia [1]. Single photon emission computerized tomography (SPECT) myocardial perfusion imaging (MPI) shows the location, extent and severity of myocardial ischemia or infarction [2–4], and is a widely used non-invasive method for the diagnosis of CAD [5, 6]. However, in patients with multivessel CAD, especially those with diffuse three-vessel disease, SPECT MPI may be falsely negative as a result of "balanced ischemia", resulting in missed diagnosis of severe CAD [7–9].

It is well known that coronary artery calcium score (CACS) is a reflection of coronary atherosclerotic plaque burden; as CACS rises, obstructive CAD is more likely to develop [10, 11]. With SPECT/CT, MPI and CACS can be performed as a single examination, giving the patient two images in one visit. Previous study shown that the addition of CACS to stress MPI improves the diagnosis of CAD [12]. However, most current studies have focused on global CACS for all epicardial vessels, rather than specific vessels, and previous study have shown that regional coronary calcification is moderately associated with regional myocardial ischemia [13]. To date, there are no comparative studies on the value of MPI combined with regional or global CACS for detecting obstructive CAD. Whether regional CACS can improve SPECT MPI to detect obstructive CAD remains unclear. Therefore, the aim of this study was to verify whether regional CACS has additional diagnostic value for obstructive CAD in suspected CAD patients, compared to SPECT MPI and global CACS.

## Methods

### Study cohort and population

**Fig. 1:**
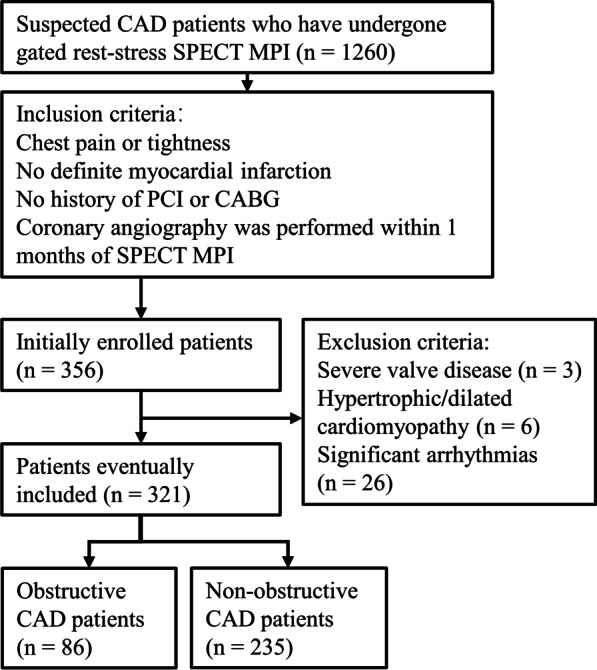
Recruitment flow chart. CAD, coronary artery disease; SPECT, single photon emission computerized tomography; MPI, myocardial perfusion imaging; PCI, percutaneous coronary intervention; CABG, coronary artery bypass grafting

In this retrospective study, we recruited suspected CAD patients who underwent gated rest-stress SPECT MPI at the Third Affiliated Hospital of Soochow University between March 2019 and October 2021. Inclusion criteria were as follows: (1) patients admitted due to chest pain or tightness, (2) no history of definite myocardial infarction, (3) no history of percutaneous coronary intervention (PCI) or coronary artery bypass grafting (CABG), (4) coronary angiography was performed within one month after the examination. Exclusion criteria were: (1) severe valvular disease, (2) hypertrophic or dilated cardiomyopathy, (3) severe arrhythmias. Finally, 321 patients were included in the study. The detailed recruitment flow chart is shown in Fig. 1. According to the guidelines [14], the pre-test probability (PTP) of CAD was calculated based on the patient's age, sex, and type of chest pain. The research protocol complied with the Declaration of Helsinki and was approved by the Ethics Committee of the Third Affiliated Hospital of Soochow University.

### SPECT MPI acquisition and analysis

Each patient underwent a 2-day rest-stress ^99m^technetium-sestamibi (^99m^Tc-MIBI) MPI. In patients without exercise stress contraindications, exercise stress MPI was performed using a modified Bruce protocol, whereas in patients with exercise stress contraindications, pharmacological stress MPI was performed using adenosine. In brief, adenosine was intravenously infused at 0.14 mg/kg min for 6 min, and the ^99m^Tc-MIBI (radiochemical purity > 95%, injected dose of 740–925 MBq) was injected at 3 min after adenosine injection. After 60–90 min, an image acquisition camera equipped with a parallel-hole collimator with low energy and high resolution (Symbia T16, Siemens Medical Systems, Erlangen, Germany) was used to capture images. With a 64 × 64 matrix and a magnification of 1.45, sixty-four images covering 180° were acquired. In order to obtain left ventricular (LV) short-axis, horizontal long-axis, and vertical long-axis images, projection data were filtered using a Butterworth filter (order 5; cutoff frequency 0.4). All image acquisition procedures follow the recommendations of the relevant guidelines [15]. CT attenuation correction was applied to all MPI.

SPECT myocardial perfusion images were analyzed using a 17-segment model [16], which separated the segments into left anterior descending coronary artery (LAD), left circumflex coronary artery (LCX), and right coronary artery (RCA) territories. All scans were independently interpreted by 2 nuclear medicine physicians with 20 years and more experience who were unaware of the CACS and coronary angiography results. A third physician would read the images if two physicians disagreed on the results, and the opinion with the most votes would be followed. Reversible myocardial ischemia was defined as decreased perfusion in two or more consecutive myocardium segments on stress MPI images and partial or complete return to normal on resting imaging.

### CACS acquisition and analysis

Following MPI acquisition, an electrocardiogram gated chest computed tomography (CT) was performed for CACS, with the following parameters: tube voltage 120 kV, tube current 100 mA, thickness 3 mm. The scan ranged from the plane below the trachea to 1–2 cm below the diaphragm surface of the heart, and the scanning time was 8–13 s after inhalation. Data were collected between 60 and 80% of the R-R interval. Agatston automatic analysis software was used to detect coronary calcifications [17], identifying areas in the coronary arteries that exceeded 130 HU. Global CACS was calculated by summing the calcification scores of left main coronary artery (LM), LAD, LCX, RCA and their main branches. LAD, LCX, and RCA regional arteries were calculated separately. The CACS for LAD was calculated as the sum of CACS of LM and LAD. The CACS for LCX was the sum of CAC scores of LM and LCX [18].

### Coronary artery angiography

Two experienced cardiologists interpreted all coronary angiograms through visual analysis. Obstructive CAD was defined as ≥ 70% narrowing in LAD, LCX, RCA or their main branches, and ≥ 50% narrowing in LM [19]. Angiograms would be read by a third senior physician if two physicians disagreed on the results, and the opinion with the most votes would be followed.

### Statistical methods

A continuous variable that conforms to the normal distribution was expressed as a mean ± SD, while a continuous variable that does not conform was expressed as a median [interquantile range: IQR]. We used unpaired t-tests or Mann–Whitney U-tests for continuous variables and chi-square tests for categorical variables to compare the two groups. Multiple groups were compared using one-way ANOVA for continuous variables with a normal distribution. Due to the wide range of regional and global CACS, the natural logarithm of CACS (LogCACS) was used in receiver operator characteristic curve (ROC) analysis. MedCalc (version 18.2.1; MedCalc Software) was used to draw and analyze the ROC. Youden index was used to determine the best cut-off value. *P* < 0.05 was considered statistically significant. Integrated discrimination improvement (IDI) and net reclassification improvement (NRI) were calculated to determine the incremental value of regional and global CACS for predicting obstructive CAD. All statistical analyses were conducted using R statistical software (version 4.1.0).

## Results

### Patient characteristics

It was found that 86 out of 321 (26.8%) suspected CAD patients had obstructive CAD. The PTP was 56.7 ± 21.3 for the obstructive CAD group and  54.1 ± 19.8 for the non-obstructive CAD group (*P* = 0.304). The proportion of males in the obstructive CAD group was higher than that in the group without obstructive CAD (70.9% vs. 57.4%, *P* = 0.028). Diabetes was more common in patients with obstructive CAD, and there were no significant differences between the two groups in terms of age, BMI, hyperlipidemia, hypertension, or smoking history. Patients with obstructive CAD had a slightly lower left ventricular ejection fraction (LVEF) than those with non-obstructive CAD, but there was no significant difference. The proportion of reversible myocardial ischemia in obstructive CAD group was significantly higher than that in non-obstructive CAD group. In addition, as shown in Table 1, the obstructive CAD group had higher CACS than the non-obstructive CAD group.

**Table 1:** Characteristics of study population

Variables	Without obstructive CAD (n = 235)	Obstructive CAD (n = 86)	*P* value
Male (%)	135 (57.4)	61 (70.9)	0.028
Age (years old)	62.2 ± 9.1	62.1 ± 10.0	0.914
BMI (kg/m^2^)	25.0 ± 3.1	25.0 ± 3.2	0.834
PTP	54.1 ± 19.8	56.7 ± 21.3	0.304
Hyperlipidemia (%)	82 (34.9)	26 (30.2)	0.434
Diabetes (%)	42 (17.9)	32 (37.2)	< 0.001
Hypertension (%)	152 (64.7)	61 (70.9)	0.294
Smoking (%)	75 (31.9)	36 (41.9)	0.097
ACEI (%)	97 (41.3)	46 (53.5)	0.051
Beta blockers (%)	138 (58.7)	54 (62.8)	0.510
Reversible myocardial ischemia (%)	76 (32.3)	45 (52.3)	0.001
LVEF (%)	62.9 ± 3.1	61.9 ± 4.6	0.055
CACS	0.0 (0.0–46.0)	75.1 (3.5–343.0)	< 0.001
CACS: 0 (%)	129 (54.9)	16 (18.6)	< 0.001
CACS: 1–100 (%)	67 (28.5)	31 (36.0)	< 0.001
CACS: 101–399 (%)	24 (10.2)	19 (22.1)	< 0.001
CACS ≥ 400 (%)	15 (6.4)	20 (23.3)	< 0.001
Coronary angiography (%)			
One vessel disease		52 (60.5)	
Two vessel disease		25 (29.1)	
Three vessel disease		9 (10.5)	

CAD, coronary artery disease; BMI, body mass index; PTP, pre-test probability; ACEI, angiotensin-converting enzyme inhibitor; LVEF, left ventricular ejection fraction; CACS, coronary artery calcium score

### Characteristics of CACS at the patient and vascular levels

**Fig. 2:**
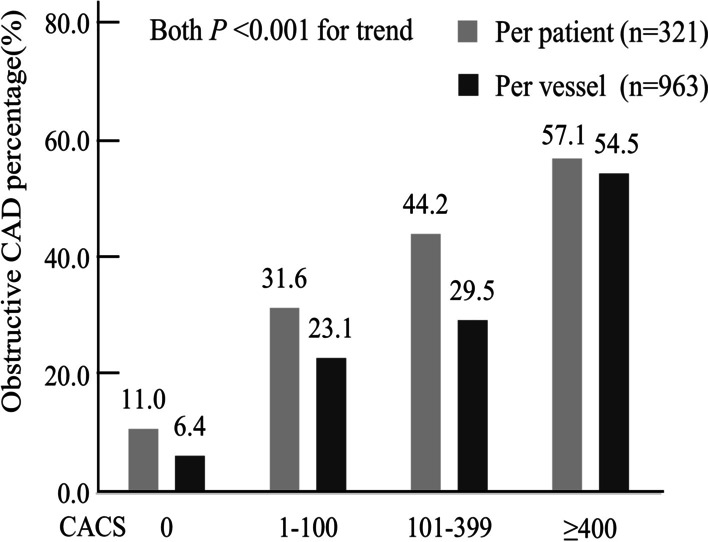
Distribution of obstructive CAD in different degrees of CACS at the patient and vascular levels. CAD, coronary artery disease; CACS, coronary artery calcium score

As shown in Table 2, 129 (13.4%) of 963 territories had severe stenosis, including 67 cases (51.9%, 67/129) in LAD, 41 cases (31.8%, 41/129) in LCX, and 21 cases (16.3%, 21/129) in RCA. We divided global and regional CACS into 4 groups: 0, 1 to 100, 101 to 399, and ≥ 400 [20]. As shown in Fig. 2, with the increase of CACS, the obstructive CAD also had an increasing trend (Both *P* for trend < 0.001). At the patient level, the proportion of obstructive CAD was 11.0% (16/145) in 145 patients with CACS = 0. In the groups with CACS 1–100, 101–399 and ≥ 400, the proportions of obstructive CAD were 31.6% (31/98), 44.2% (19/43), and 57.1% (20/35), respectively. At the vascular level, of the 963 territories in 321 patients, 653 had a regional CACS of 0, of which 6.4% (42/653) had obstructive CAD. In the territories with CACS 1–100, 101–399 and ≥ 400, the proportions of obstructive CAD were 23.1% (46/199), 29.5% (23/78), and 54.5% (18/33).

**Table 2:** Characteristics of MPI and CACS at the vascular level (n = 963)

Variables	Without obstructive CAD (per vessel n = 834)	Obstructive CAD (per vessel n = 129)	*P* value
Reversible myocardial ischemia (%)	113 (13.5)	49 (38.0)	< 0.001
CACS: 0 (%)	611 (73.3)	42 (32.6)	< 0.001
CACS: 1–100 (%)	153 (18.3)	46 (35.7)	< 0.001
CACS: 101–399 (%)	55 (6.6)	23 (17.8)	< 0.001
CACS ≥ 400 (%)	15 (1.8)	18 (14.0)	< 0.001
Coronary angiography (%)			
LAD territory		67 (51.9)	
LCX territory		41 (31.8)	
RCA territory		21 (16.3)	

MPI, myocardial perfusion imaging; CAD, coronary artery disease; CACS, coronary artery calcium score; LAD, left anterior descending coronary artery; LCX, left circumflex coronary artery; RCA, right coronary artery

Among 834 territories without obstructive CAD, 73.3% (611/834) had CACS equal to 0. In 129 territories with obstructive CAD, 67.4% (87/129) had different degrees of coronary artery calcification. In addition, the proportion of reversible myocardial ischemia in territories with obstructive CAD was significantly higher than that without obstructive CAD (38.0% vs. 13.5%, *P* < 0.001).

### Global CACS and regional CACS for predicting obstructive CAD

**Fig. 3:**
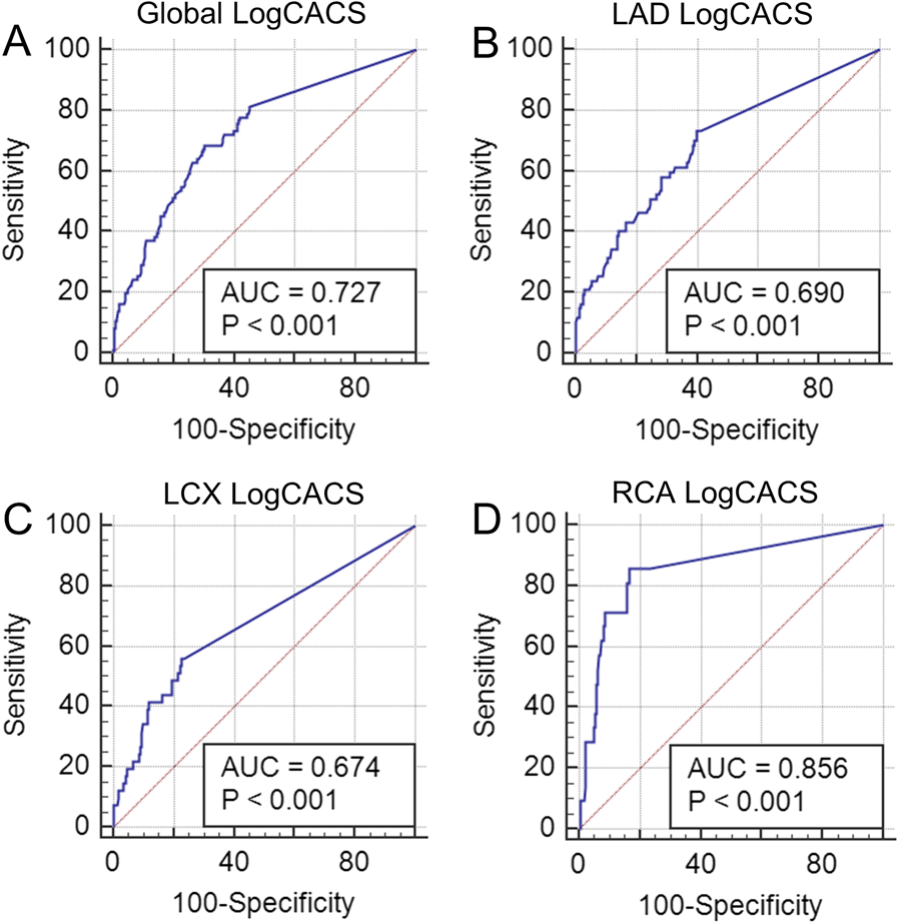
ROC analysis of regional and global CACS for detecting obstructive CAD. **A** ROC analysis of global LogCACS for detecting obstructive CAD. **B** ROC analysis of regional LogCACS for detecting obstructive CAD in LAD. **C** ROC analysis of regional LogCACS for detecting obstructive CAD in LCX. **D** ROC analysis of regional LogCACS for detecting obstructive CAD in RCA. ROC, receiver operator characteristic curve; CAD, coronary artery disease; CACS, coronary artery calcium score; LAD, left anterior descending coronary artery; LCX, left circumflex coronary artery; RCA, right coronary artery; AUC, the area under the receiver operator characteristic curve

We performed ROC analysis separately for global CACS and regional CACS for predicting obstructive CAD. As shown in Fig. 3, the AUC of global LogCACS was 0.727 (95%CI 0.674–0.775, *P* < 0.001), and the best cut-off value was 1.41 (unlog-transformed: 25.70). The AUC of regional LogCACS for LAD territories was 0.690 (95%CI 0.636–0.740, *P* < 0.001), and the best cut-off value was 0.23 (unlog-transformed: 1.70). The AUC of regional LogCACS for LCX territories was 0.674 (95%CI 0.619–0.725, *P* < 0.001), and the best cut-off value was 0.20 (unlog-transformed: 1.58). The AUC of regional LogCACS for RCA territories was 0.856 (95%CI 0.813–0.892, *P* < 0.001), and the best cut-off value was 0.93 (unlog-transformed: 8.51). Regional CACS had a better diagnostic performance in RCA territories.

### MPI combined with global and regional CACS for predicting obstructive CAD

**Fig. 4:**
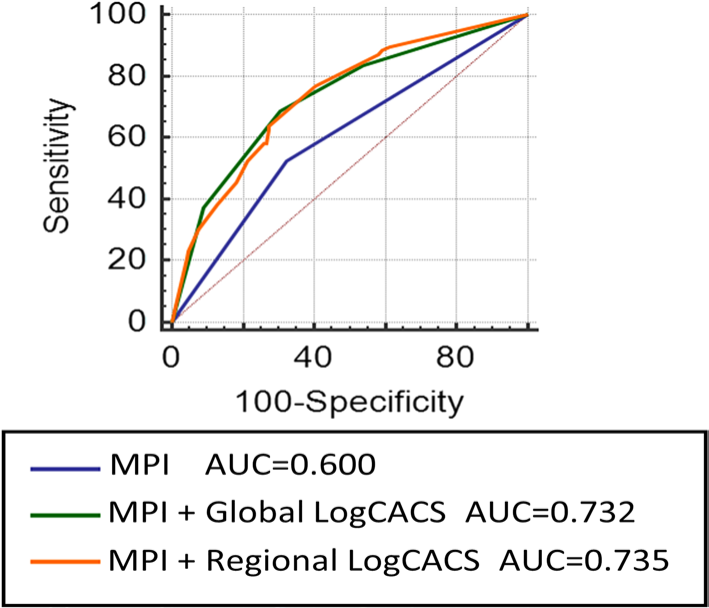
ROC analysis of global and regional CACS for detecting obstructive CAD based on MPI. ROC, receiver operator characteristic curve; CAD, coronary artery disease; CACS, coronary artery calcium score; MPI, myocardial perfusion imaging; AUC, the area under the receiver operator characteristic curve

**Fig. 5:**
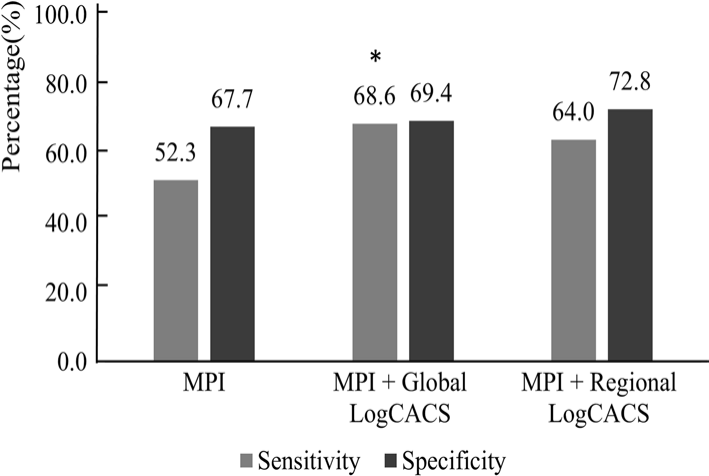
The sensitivity and specificity of MPI and combination of MPI and CACS for detecting obstructive CAD. MPI, myocardial perfusion imaging; CAD, coronary artery disease; CACS, coronary artery calcium score. *P < 0.05 (compared to MPI)

To demonstrate the value of CACS compared to MPI, we established three models: Model 1, MPI; Model 2, MPI + global LogCACS; Model 3, MPI + regional LogCACS. The combined models were constructed by logistic regression, and the probability of predicting obstructive CAD was calculated for the three models. The ROC was plotted by probability values, and the diagnostic effectiveness of three models was compared. As shown in Fig. 4 and Table 3, the AUCs for Model 1, Model 2 and Model 3 were 0.600 (95% CI 0.544–0.654, *P* < 0.001), 0.732 (95% CI 0.680–0.780, *P* < 0.001) and 0.735 (95% CI 0.683–0.782, *P* < 0.001), respectively. When global LogCACS was added, the AUC of Model 2 was significantly higher than Model 1 (0.732 vs. 0.600, *P* < 0.001). Similar results occur in regional CACS, where the AUC of Model 3 was higher than Model 1 (0.735 vs. 0.600, *P* < 0.001). However, the AUC of Model 3 was not significantly higher than Model 2 (0.735 vs. 0.732, *P* = 0.898). The sensitivity and specificity of Model 2 and Model 3 were calculated according to the best cut-off value and compared with Model 1, which are shown in Fig. 5 and Table 4.

**Table 3:** Comparison of three models for identification of obstructive CAD

Models	AUC (95% CI)	Difference in AUC (95% CI)	*P*	NRI (95% CI)	*P*	IDI (95% CI)	*P*
Model 1	0.600 (0.544–0.654)	Reference	–	Reference	–	Reference	–
Model 2	0.732 (0.680–0.780)	0.132 (0.068–0.196)	< 0.001	0.580 (0.410–0.749)	< 0.001	0.117 (0.081–0.153)	< 0.001
Model 3	0.735 (0.683–0.782)	0.135 (0.070–0.199)	< 0.001	0.619 (0.433–0.806)	< 0.001	0.108 (0.071–0.146)	< 0.001
Model 2	0.732 (0.680–0.780)	Reference	–	Reference	–	Reference	–
Model 3	0.735 (0.683–0.782)	0.003 (− 0.039 to 0.045)	0.898	0.040 (− 0.108 to 0.188)	0.598	− 0.009 (− 0.039 to 0.022)	0.576

CAD, coronary artery disease; AUC, the area under the receiver operator characteristic curve; NRI, net reclassification index; IDI, integrated discrimination improvement. Model 1, MPI; Model 2, MPI + global LogCACS; Model 3, MPI + regional LogCACS

**Table 4:** The diagnostic efficacy of MPI alone and combination of MPI and CACS for detecting obstructive CAD in suspected CAD patients (n = 321)

	MPI	MPI + Global CACS	MPI + Regional CACS
Sensitivity (%)	52.3	68.6	64.0
Specificity (%)	67.7	69.4	72.8
PPV (%)	37.2	45.0	46.2
NPV (%)	79.5	85.8	84.7
Accuracy (%)	63.6	69.2	70.4

MPI, myocardial perfusion imaging; CAD, coronary artery disease; CACS, coronary artery calcium score; PPV, positive predictive value; NPV, negative predictive value

**Fig. 6:**
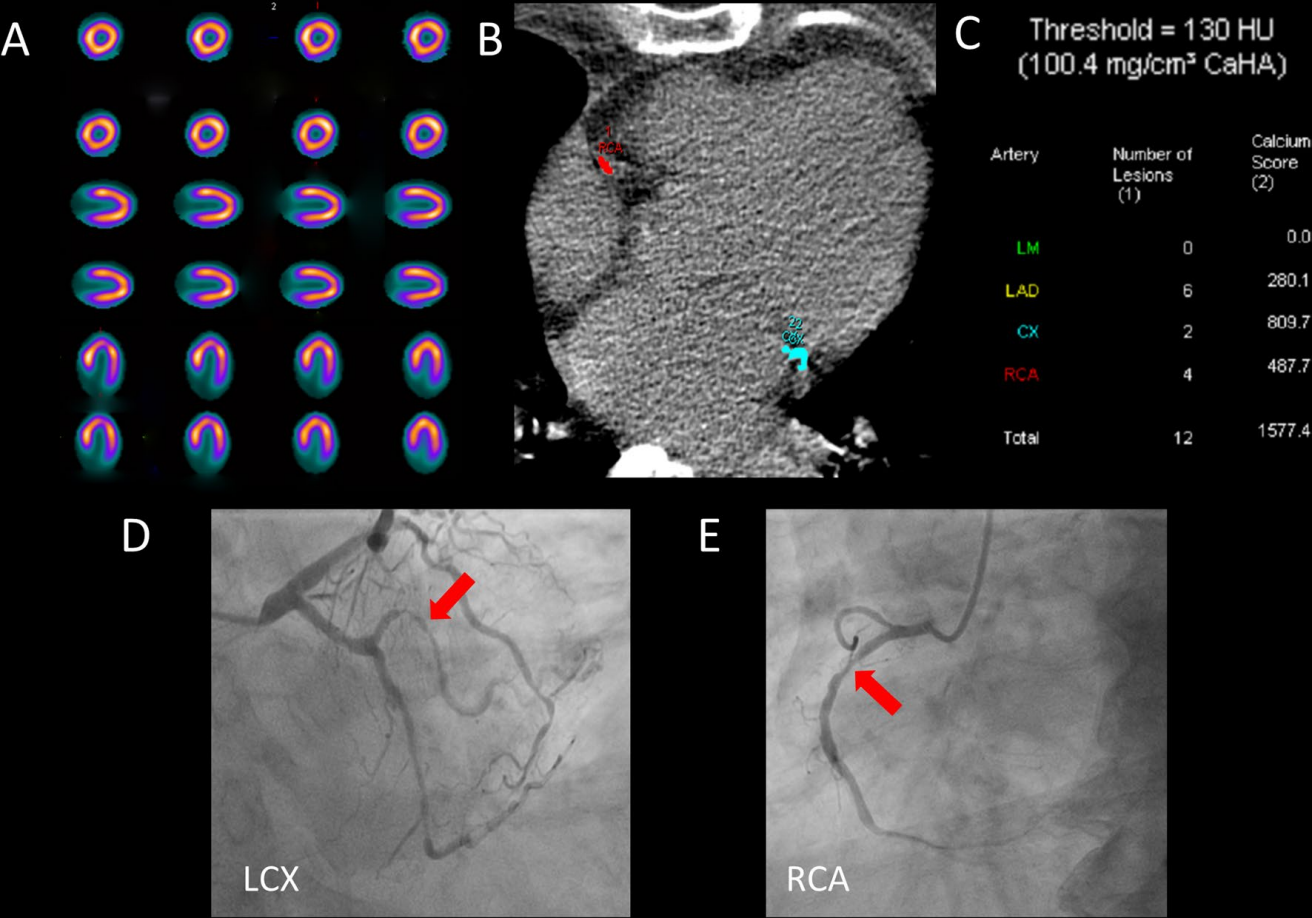
Patient example 1. A 71-year-old woman with hypertension and diabetes presented with chest pain after exercise for 1 year and was admitted for examination. **A** There are no abnormalities in rest-stress MPI. **B** CACS quantitative analysis was performed by Agatston software. **C** Regional CACS: LAD: 280.1; LCX: 809.7; RCA: 487.7; Global CACS:1577.4. **D**, **E** Coronary angiography: LM: negative; LAD: 40–50% proximal stenosis, 60% middle and distal stenosis, LCX: 60–80% distal stenosis, RCA: 70% proximal stenosis. This patient had extensive calcification in three coronary arteries, which was highly suggestive of obstructive CAD even if the rest-stress MPI was negative

**Fig. 7:**
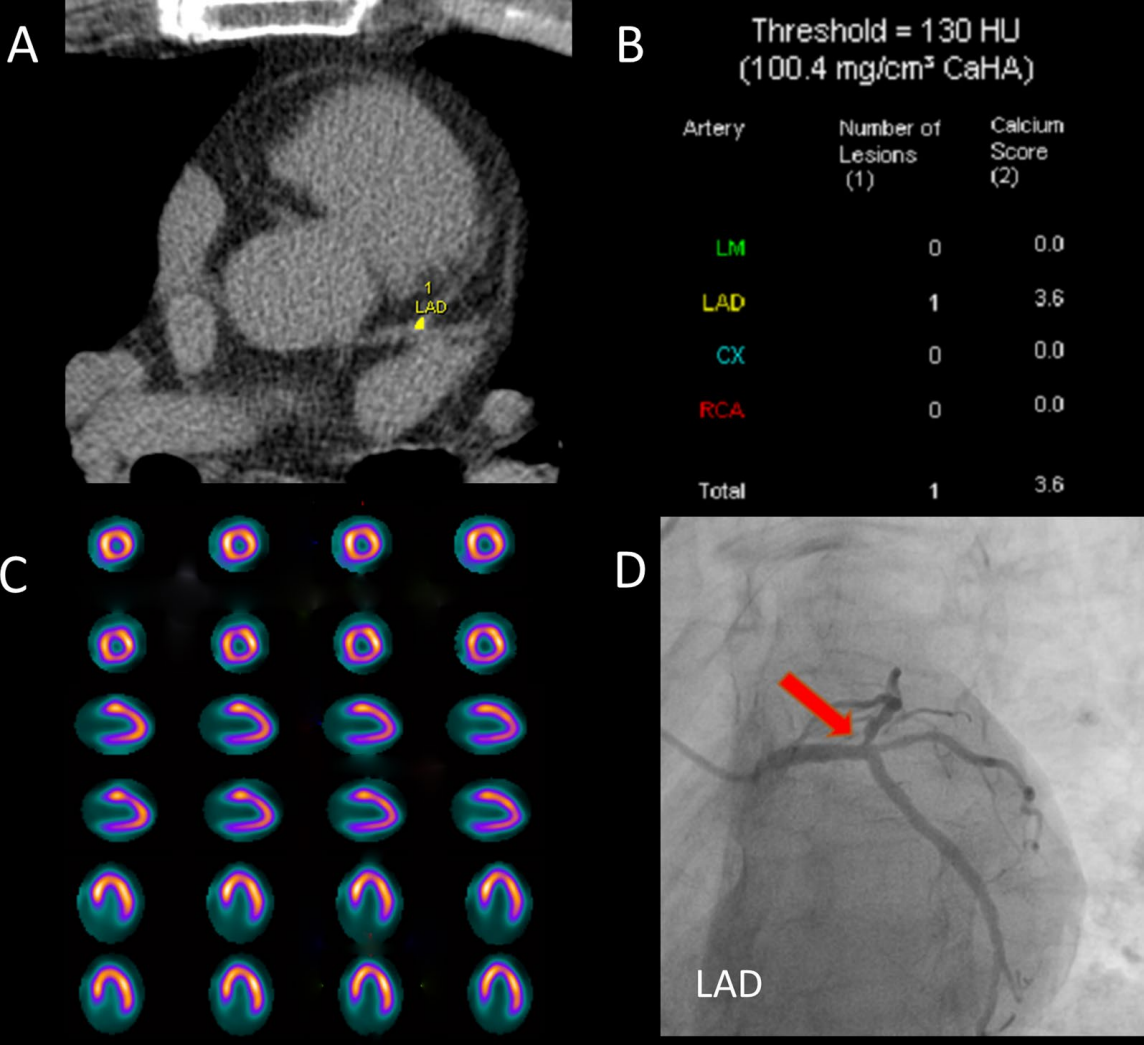
Patient example 2. A 65-year-old male patient without hypertension or diabetes was admitted to the hospital with chest pain for 3 months after exercise. **A** CACS quantitative analysis was performed by Agatston software. **B** Regional CACS: LAD: 3.6 (log-transformed value: 0.556), LCX: 0, RCA: 0, Global CACS: 3.6. **C** There are no abnormalities in rest-stress MPI. **D** Coronary angiography: LM: 30% distal stenosis, LAD: 75% open stenosis, LCX: 40–50% distal stenosis, RCA: 30% proximal and middle stenosis. The regional CACS for LAD exceeded the best cut-off value, while the global CACS did not, which was consistent with the results of coronary angiography, reflecting the value of regional CACS

Compared to Model 1, the NRI for Model 2 was 0.580 (95% CI 0.410–0.749, *P* < 0.001) and the integrated discrimination improvement IDI was 0.117 (95% CI 0.081–0.153, *P* < 0.001). When regional CACS was added to Model 1, Model 3 had an NRI of 0.619 (95% CI 0.433–0.806, *P* < 0.001) and an IDI of 0.108 (95% CI 0.071–0.146, *P* < 0.001), as shown in Table 3. In addition, regional CACS did not appear to provide a significant gain in detecting obstructive CAD compared to global CACS (NRI: 0.040, *P* = 0.598; IDI: − 0.009, *P* = 0.576). Figures 6 and 7 show two cases.

## Discussion

This study showed that patients with obstructive CAD tended to increase with the increase of global and regional CACS. Regional CACS had a better diagnostic performance in RCA territories (AUC 0.856, *P* < 0.001). The AUC of combined regional LogCACS and MPI was significantly higher than that of MPI alone (0.735 vs. 0.600, *P* < 0.001). However, on the basis of MPI, the AUC of combined regional CACS was not significantly higher than that of global CACS (0.735 vs. 0.732, *P* = 0.898). The sensitivity and specificity of regional CACS combined with MPI for detecting obstructive CAD were 64.0% and 72.8%.

It is widely accepted that SPECT MPI is an evidence-based, noninvasive method that can detect myocardial ischemia and obstructive CAD [4, 21, 22]. In general, the sensitivity of exercise stress SPECT MPI for the diagnosis of CAD was 82–88%, and the specificity was 70–88%; the sensitivity of pharmacologic stress SPECT MPI for the diagnosis of CAD was 88–91%, and the specificity was 75–90% [23]. Schepis et al. [12] discovered a significant improvement in the sensitivity for detecting CAD when CACS was combined with stress MPI (76% vs. 86%); however, the specificity was not affected by the combination (91% vs. 86%). The present study confirmed the incremental value of regional and global CACS for diagnosing obstructive CAD compared to SPECT MPI. The sensitivity and specificity of MPI combined global and regional CACS were 68.6% and 69.4%; 64.0% and 72.8%, respectively. In this study, the AUC of MPI for diagnosing obstructive CAD was low (0.600). This may be related to the following reasons: (1) some patients have microvascular disease, resulting in abnormal perfusion, normal angiography or mild stenosis, (2) in the category 50–70% stenosis, 35% were functionally significant, in the category 71–90% stenosis, 20% were not functionally significant [1], (3) patient selection bias.

Brodov et al. [18] found that MPI combined with CACS based on ^82^Rb PET/CT could improve the diagnosis of obstructive CAD. The AUC of MPI combined with regional CACS was 0.85, higher than that of MPI (AUC 0.81). Similar to our study, there was no significant difference between global and regional CACS in detecting obstructive CAD. However, different from their research, due to the lower distribution and extent of coronary artery calcification in Asian populations [24], we sought to find the best cut-off values of regional CACS in each territory. In addition, SPECT MPI is currently more widely used in China than PET/CT. Evaluation of regional and global CACS based on SPECT MPI may have broader application prospects. This study showed that regional CACS had the highest diagnostic efficiency in the RCA territory. It may be related to the relatively low number of lesions in RCA territories or not affected by LM on the results of CACS and coronary angiography, which needs further study.

The pathogenesis and histopathology of coronary atherosclerosis have been thoroughly examined, and coronary artery calcium has been confirmed to occur at the site of coronary atherosclerosis, which results from the development of coronary atherosclerosis to a certain degree [12]. However, a high CACS does not mean that the coronary artery where it is located will definitely be severely narrowed. During the course of atherosclerosis, coronary arteries may undergo compensatory remodeling and dilation, resulting in normal or mild stenosis on coronary angiography [25]. A calcium score of 0 does not necessarily mean that the coronary artery is not narrowing. The above mechanism may refer to the early coronary atherosclerotic process, where the coronary plaque is composed of lipids or fibers and lacks obvious calcification [26]. Furthermore, voxels with a measurement area of at least 1 mm^2^ and a density of at least 130 HU are considered calcifications by the Agatston algorithm, so microcalcifications below this threshold may be ignored [27]. As shown in this study, obstructive CAD was still present in 11% of patients with a CACS = 0, and 42.9% of patients with a CACS ≥ 400 had no significant coronary stenosis.

According to the latest ESC guidelines [14], MPI is a class I recommendation for suspected CAD patients. Low-dose chest CT evaluation of coronary artery calcification is also commonly used for CAD screening noninvasively. Our study showed that regional CACS was not superior to the global CACS in detecting obstructive CAD based on MPI. The reason may be related to the small number of obstructive CAD patients in this study, and further large sample study is needed for verification. On the other hand, it was suggested that both regional and global CACS can be used to detect obstructive CAD, which extends the application of CACS in SPECT MPI.

### Limitation

Several limitations of this study should be considered. Firstly, the outcomes used in the study were still anatomic based coronary angiography, lacking hemodynamic criteria such as FFR. Secondly, all enrolled patients underwent coronary angiography, and referral bias may exist. Thirdly, the distribution and extent of coronary calcification in Asian populations are lower than those in Western countries, and the findings need to be validated in multiracial populations. In addition, the sample size of this study was small and needed further validation in a prospective, large sample, multicenter study.

## Conclusions

Regional CACS was effective in detecting obstructive CAD in RCA territory. Based on SPECT MPI, regional CACS improved the detection of obstructive CAD, but was not superior to global CACS.

## Data Availability

The datasets used during the current study are available from the corresponding author on reasonable request.

## Abbreviations

CAD: Coronary artery disease
SPECT: Single photon emission computerized tomography
MPI: Myocardial perfusion imaging
CACS: Coronary artery calcium score
PCI: Percutaneous coronary intervention
CABG: Coronary artery bypass grafting
LV: Left ventricular
LAD: Left anterior descending coronary artery
LCX: Left circumflex coronary artery
RCA: Right coronary artery
LM: Left main coronary artery
LVEF: Left ventricular ejection fraction
